# Fungal biology in the post-genomic era

**DOI:** 10.1186/s40694-014-0007-6

**Published:** 2014-10-14

**Authors:** Claudio Scazzocchio

**Affiliations:** 1 Department of Microbiology, Imperial College, London, SW7 2AZ UK; 2 Institut de Génétique et Microbiologie, CNRS UMR 8621, Université Paris-Sud, Orsay, 91405 France

## Abstract

In this review I give a personal perspective of how fungal biology has changed since I started my Ph. D. in 1963. At that time we were working in the shadow of the birth of molecular biology as an autonomous and reductionistic discipline, embodied in Crick’s central dogma. This first period was methodologically characterised by the fact that we knew what genes were, but we could not access them directly. This radically changed in the 70s-80s when gene cloning, reverse genetics and DNA sequencing become possible. The “next generation” sequencing techniques have produced a further qualitative revolutionary change. The ready access to genomes and transcriptomes of any microbial organism allows old questions to be asked in a radically different way and new questions to be approached. I provide examples chosen somewhat arbitrarily to illustrate some of these changes, from applied aspects to fundamental problems such as the origin of fungal specific genes, the evolutionary history of genes clusters and the realisation of the pervasiveness of horizontal transmission. Finally, I address how the ready availability of genomes and transcriptomes could change the status of model organisms.

**Electronic supplementary material:**

The online version of this article (doi:10.1186/s40694-014-0007-6) contains supplementary material, which is available to authorized users.

## Looking back in awe

*We enter the post-genomic era when it is simpler and cheaper to re-sequence a whole genome to identify a point mutation rather than follow the classical path of genetic mapping and subsequent gene cloning and sequencing. We are already there.*

I started working with fungi in the autumn of 1963, which I realise with horror is already more than fifty years ago. It was not my intention to work with a fungus, but to work on the control of gene expression in any appropriate organism. I had discovered the operon model through improbable circumstances that took me to the International Congress of Biochemistry in Moscow in 1961, where François Jacob gave one of the main lectures. What fascinated me and determined my subsequent scientific quest was the logical process by which a regulation circuit could be constructed through dominance and epistasis relationships; the formal correctness of the circuit being independent of its material basis.

Even more improbable circumstances landed me in Cambridge, where John Pateman and David Cove were investigating the regulation of nitrate assimilation in *Aspergillus nidulans,* two of a handful of scientists working within the Jacob-Monod framework in a eukaryotic organism. I have recently written about those early days [1],[2].

My fellow research student Andy Darlington and I were given the task of investigating purine utilisation and its regulation. The link with the work of Pateman and Cove was that two key enzymes of nitrate and purine assimilation share the molybdenum containing cofactor they had recently discovered [3]. In 1965 I published my first fungal article, which was also my first article on purine assimilation in *A. nidulans* [4]. This year, I was a co-author of an article also dealing with purine assimilation in this same organism [5]. Sticking for over fifty years to the same problem and organism seems, by almost any standard, a case of obsessional fidelity. I did work on many other subjects in those fifty odd years, but almost without exception, I stuck with *A. nidulans* as a model organism. For the 1965 article, we isolated, by random mutagenesis, mutations in the intracellular steps of the conversion of purines to ammonium, including those in the positively-acting transcription factor. The techniques used in this and a few following articles were those of classical genetics and relatively unsophisticated biochemistry, complemented occasionally by immunology [4],[6]-[8]. For the 2014 article we sequenced some of the mutations described in 1965 and 1968, after amplifying the cognate genes by PCR, we inactivated a few genes by homologous recombination, we constructed GFP fusions to investigate the cellular localisation of the enzymes we first assayed in 1963–68 [4],[6], we investigated the pathway in a whole order of fungi (the Eurotiales), we uncovered paralogues of unknown specificity for some of the purine utilisation enzymes, we established that within the Eurotiales the hydrolysis of allantoic acid is usually catalysed by the classical allantoicase but occasionally by a completely different enzyme [9], whose cognate gene was almost certainly horizontally transferred from bacteria [5].

I have indulged in this personal reminiscence to contrast the technologies used in 1963–65 with those of 2014: Gene cloning, PCR, fungal reverse genetics, DNA sequencing, GFP fusions allowing the study of protein localisation *in vivo*, the ready availability of structures for orthologues of the enzymes we were studying, which allowed educated guesses on paralogue specificities, and last but not least, the public availability of hundreds of fungal (and thousands of bacterial) genomes which allow one to speculate on more or less probable evolutionary scenarios.

All these possibilities were undreamt off when I started my Ph. D. The first time I heard about DNA being the genetic material was in a series of lectures given in 1958 by Hans Tuppy, a co-worker of Fred Sanger on the sequencing of the insulin molecule, who went on to sequence the peptidic hormone oxytocin and later cytochrome C. This expert on protein sequencing pondered whether we would unravel one day the genetic code by sequencing both proteins (which could be done at the time with lots of work) and DNA. He was very pessimistic about the latter.

In 1963, the reduction of the classical gene to its molecular avatar was almost complete. This accomplishment could be called the first revolution in molecular biology, or better still the scientific revolution that gave birth to molecular biology. We could date this process from the first article pertaining to the one gene-one enzyme concept [10] and the establishment of DNA as the determinant of capsular antigens in *Diplococcus pneumoniæ* [11] to the establishment of co-linearity between genes and proteins [12],[13], not forgetting the convergence of the genetic [14] and biochemical approaches to the deciphering of the genetic code [15].

A few details were missing. We were working within the framework of what Crick called in 1959 the central dogma [16]. We knew that the genes we worked with were DNA; we knew they encoded proteins, we knew the code; however we could not access or manipulate the genes directly. The techniques available were still those of classical genetics. The molecular reduction of the gene was in the conceptual background, not in the operations we performed. It was a persistent ghost, not a helpful jinni. In 1968, Gunther Stent published an article, “That was the Molecular Biology, that was” [17] which is related in more than one way to the concept of the End of History. I will not discuss here this article in detail, but in a nutshell Stent proclaimed the end of molecular biology. He stated that all we had to do was to iron out details, dot the Is, as they say. Little did he know.

The second revolution in Molecular Biology started about 1973 and it is still with us. While the first revolution borrowed concepts [18] and mainly techniques from *outside* the field (ultracentrifugation, electrophoresis, chromatography, X ray diffraction), this second revolution took root in developments *within* the field. Restriction enzymes, ligases, reverse transcriptases, DNA polymerases, allowed the jinni to escape from the jar, that is, to intervene directly on the structure of the genetic material. The epistemological consequence of this second revolution was to deconstruct the isomorphism between the formal gene and its molecular substratum, the DNA sequence. This is another story, which I hope to discuss in detail elsewhere. It also completed the conceptual unification of the biological sciences initiated with the rediscovery of Mendel’s laws in 1900. It had the unforeseen consequence of transforming research in Molecular Biology from a convivial, though intensive, labour-light discipline into an autistic, thought-light, labour intensive pursuit.

This second revolution entered the fungal research community with the establishment of transformation techniques for *Saccharomyces cerevisiæ* [19],[20]. This early technical development is at the basis of the hegemony of the *S. cerevisiæ* research community, which could, on its own, constitute an interesting chapter of the sociology of science. Transformation of three other model organisms *Neurospora crassa* [21], *Schizosaccharomyces pombe* [22] and *A. nidulans* [23],[24] followed.

There is a paradox underlying what I have called the hegemony of *S. cerevisiæ*. The success of *S. cerevisiæ* as a model is not based on its similarities with other eukaryotes, but on its differences. One could even say, from an “eukaryotist” point of view, on its deficiencies. It has easily available replication origin sequences, which as we readily learnt to our chagrin, do not function in other eukaryotes. It has an autonomous nuclear plasmid. It shows no heterologous recombination, easily allowing gene replacement procedures. Last but not least its strikingly tiny centromeres allow the engineering of single-copy stable plasmids. Workers with other organisms had to struggle mightily to offset the eukaryotic perfection of their models (see for example [25]-[28].

The third revolution started more quietly, almost unannounced. A forerunner of what was to come was the determination of the sequence of the 5′ of the *lacZ* mRNA, all 39 nt of it [29]. The first whole, “massive” sequences come from Sanger’s lab; bacteriophage ΦX174 (5375 nt, [30], human mitochondrial DNA (16569 bp, [31]. Our modest contribution to the not yet born science of genomics was the almost complete sequence of the *A. nidulans* mitochondrial DNA (app 34 kb [32] ^a^.

The early history of whole organism genome sequencing, from *Hæmophilus influenzæ* in 1995, *Saccharomyces cerevisiæ* in 1996, *Cænorhabditis elegans* in 1998, *Drosophila melanogaster* in 1999, *Arabidopsis thaliana* in 2000 to the public announcement of the human draft genome in 2000 (http://www.youtube.com/watch?v=slRyGLmt3qc) is too well known to be repeated here. For a time, the completion of every single genome led to public announcements to the press, editorials in Science and/or Nature, each genome was a scientific and mediatic event. No longer so. These genomes were sequenced by variations of the Sanger di-deoxy method. It looked at the time as though only the genome of a few model organisms would be obtained, and this in turn would reinforce their use as models. I remember a meeting in 1996 where we argued heatedly whether we should go for the genome sequencing of *A. nidulans* or *Neurospora crassa*.

What are called, “next generation” sequencing methods, depart in different ways from the Sanger procedures. What is important here is that their implementation diminished from about 2008 the cost and time scale of whole genome sequences by orders of magnitude [33]. An NIH site shows a graph recording the cost per megabase from about 5292 $US in 2001 to about 5 cents in 2013, or using a different parameter, the cost of sequencing a single human genome, from slightly under 100 million $US in 2001 to about 5000 $US in 2013 (http://www.genome.gov/sequencingcosts/).

There are at the time of writing 384 complete fungal genomes at http://genome.jgi.doe.gov/fungi/fungi.info.html, increasing almost by the hour. The *Saccharomyces* database contains genomes of 28 different strains of *S. cerevisiæ*. We are getting to the point that if you isolate a new strain of a fungus, let alone a new species, the first thing you do is to get its genome sequenced. Massive parallel sequence techniques also led to the development of RNAseq, by which we can, together with the genome, know the transcriptome; and this in several growth conditions or developmental stages (see for fungal examples [34],[35].

At the onset of the genomic revolution the selection of the organisms to be sequenced was guided by their status as model systems, the exception being that the human genome was obtained before the mouse one, which was surely a political rather than a scientific choice. There followed, before the crucial date of 2008, organisms that were important as pathogens or because of their industrial applications (eg. *Candida albicans* and *Aspergillus fumigatus* among the former *Aspergillus niger* and *Phanerochæte chrysosoporium* among the latter).

Among all the present and foreseeable consequences of the second phase of the genomic revolution (from the inflexion point of 2008), there is one which I cannot help mentioning. More and more genomes are becoming available not because they have behind them huge research communities or industrial or medical lobbies but because they represent crucial nodes in the tree of life. Thus we have available the genome of the sea squirt *Ciona intestinalis*, the only extant member of the placozoa (*Trichoplax adherens*) of a coral, of a comb-jelly, of a sponge, of the Coelacanth, of the Platypus. A specific programme, “Origins of multicellularity” is aimed at obtaining full genomes at the root of the opisthokonta (animals and fungi plus sister groups) with already available genomes of choanoflagellata, filasterea, icthyosporea, apusozoa, (http://www.broadinstitute.org/annotation/genome/multicellularity_project/MultiHome.html). Thus we can build phylogenies based not only on a few transcribed gene differences, but on whole concatenation of sequences, genome organisation, synteny and intron-exon organisation.

It will be impossible to give a complete and systematic account about how the post-genomic revolution is changing and will change fungal biology: I simply try to give a few examples, which have caught my interest and imagination, necessarily these choices will be somewhat subjective and arbitrary.

### Genome inspired biotechnology: enzymes

Fungi have been used for a long time as sources of extracellular (and in some cases intracellular) enzymes. The availability of whole genomes allows the search of enzymes with enhanced properties or altered specificities. Obvious examples are enzymes related to cellulose, chitin and lignin degradation. To identify enzymes with new, promising specificities, the availability of structures, or, as a second best, structural models, are of paramount importance. The relative dearth of protein structures is a limiting factor. There are more than 100,000 protein structures publically available, as compared to 175 twenty years ago. However, the methodologies to obtain them, while improving steadily, with a clear upturn about 1993, have not undergone a similar revolutionary change to that embodied by “next generation” sequencing methods (http://www.proteinstructures.com/Structure/Structure/proteinstructure-databases.html).

**Figure 1:**
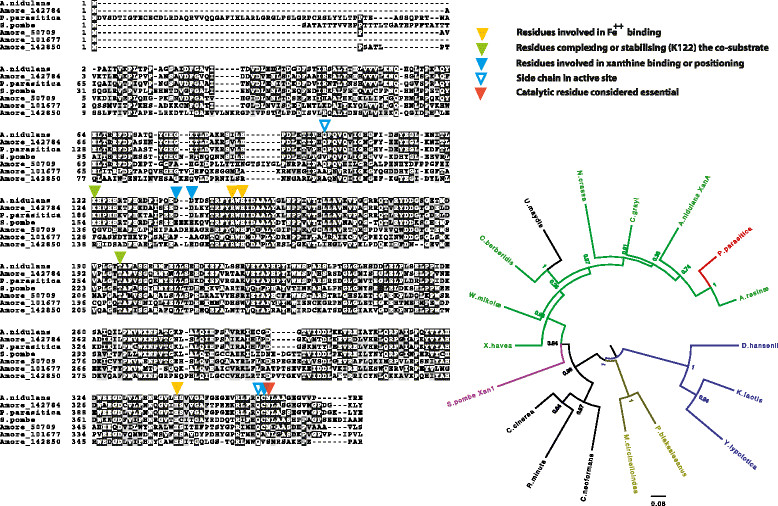
**Divergence and horizontal transmission of XanA homologues.** Left panel: Alignment of the paralogues of *Amorphotheca resinæ* (labelled Amore plus the accession number in the JGI database) with the characterised enzymes of *A. nidulans* and *S. pombe*[36],[37],[40]. Amore_142784 is the orthologue of XanA, note strict conservation of all functional residues only for this paralogue among *A. resinæ* sequences. The putative orthologue of *Phytophthora parasitica* (gi|568015616|gb|ETL89793.1) also shows conservation of all functional residues. The Fe^++^ binding residues are, as expected, conserved in all paralogues. Alignment carried out with MAFT (G-INS-i) visualisation with Box-shade. Right panel: A maximum likelihood rooted tree of putative orthologues of XanA representing different fungal taxons. Green: Ascomycetes, Pezizmycotina, Blue: Ascomycetes, Saccharomycotina, Purple, Ascomycetes, Taphrinomycotina. Olive green: Mucoromycotina, Black: Basidiomycota. Red: *P. parasitica*, Oomycetes. Note the anomalous position of *U. maydis*. The conservation of crucial residues together with the position of *P. parasitica* within the Pezizomycotina is a clear mark of horizontal transmission. *R. minuta*, *Rhodotorula minuta*, Pucciniomycotina; *U. maydis*, *Ustilago maydis*, (Ustilaginomycotina); *C. cinerea, Coprinopsis cinerea*, *C. neoformans, Cryptococcus neoformans*, (Agaricomycotina); *A. resinæ*, *Amorphotheca resinæ* (Leotiomycetes); *A. nidulans, Aspergillus nidulans*, (Eurotiomycetes); *C. grayi, Cladonia grayi,* (Lecaranomycetes); *N. crassa, Neurospora crassa*, (Sordariomycetes); *C. berberidis, Curcubitaria berberidis*, (Dothideomycetes); *W. mikolæ, Wilcoxina mikolæ* (Pezizomycetes), *S. pombe, Schizosaccharomyces pombe*, (Taphrinomycotina); *P. blakesleeanus, Phycomyces blakesleeanus, M. circinelloindes* (Mucoromycotina), *D. hansenii, Debaromyces hansenii*, *K. lactis, Kluyveromyces lactis*, *Y. lipolytica, Yarrowia lipolytica* (Saccharomycotina). We have included species where some experimental work was extant [36], and in other cases we chose the closest homologue to XanA within the taxon. Alignments carried out with MAFT (G-INS-i, ), http://mafft.cbrc.jp/alignment/server/ , Curation with BMG1 [41], both with defaults parameters, tree generated with PhyML [42], digits in nodes are aLRTs (Approximate Likelihood ratio test [43]. Circular tree redrawn with Figtree (http://tree.bio.ed.ac.uk/software/figtree/).

To draw an example from our recent work, we have identified a uniquely fungal enzyme, xanthine α-ketoglutarate dependent dioxygenase (XanA, [36],[37]) Genes encoding this enzyme are present as an alternative or in addition to the classical M0CO (molybdenum cofactor-containing xanthine dehydrogenase, which is universally conserved). In the genomes of *Penicillia*, but not of *Aspergillus,* we have identified paralogues which almost certainly have a different substrate specificity [5]. As dioxygenases are known to breakdown aromatic compounds, including herbicides [38], a broad investigation of these paralogue specificities would be of interest. The ascomycete *Amorphotheca* (*Hormoconis*, *Cladosporium*) *resinæ* has been isolated as a contaminant of jet fuel. It both degrades and it produces hydrocarbons. It obviously has some extraordinary metabolic capabilities [39]. A genomic search revealed four paralogues of XanA (as opposed to the standard one in most members of the Pezizomycotina). One paralogue is the obvious orthologue of XanA. The other three paralogues are necessarily Fe^++^ oxygenases as the iron binding site is conserved, but the substrate binding residues are not (Figure 1, left panel). This may provide an interesting insight into the hydrocarbon degrading enzymes of this organism. While the genome has been available for some time, to date no genomic-based research has been published for this organism.

The cytochrome P450 monoxygenase superfamily (CYP) members are involved in many different steps in both primary and secondary metabolism including the biosynthesis of ergosterol. Lanosterol 14 α-demethylase (CP51), a P450 enzyme is the target of azole antifungals (see below). The P450 superfamily has been surveyed in 47 completed fungal genomes suggesting a complex pattern of gene duplication and loss with where numbers of cognate genes varying from one (*Eremothecium cymbalariæ*) to 153 (*Aspergillus flavus*), distributed among 15 phylogenetic clades [44]. While this work provides an insight into the evolution of this superfamily in the fungi, it does not provide many clues as to new substrate specificities. A number of structures for P450 proteins are available in the RCSB PDB databank, and one can imagine docking studies, combined with high throughput methods in which the activity of enzymes expressed from constitutive promoters is assayed for libraries of substrates. This is quite feasible as throughput assays of P450 activities are available.

### Genome inspired biotechnology: secondary metabolites

Secondary metabolites made by fungi range from the providential (β-lactam antibiotics) to the fiendish (aflatoxin). Many secondary metabolites are non-ribosomal peptides or polyketides, and moreover the genes involved in their synthesis are clustered (see following sections). Thus it is relatively simple to recognise in genomes those clusters involved in their synthesis, as they usually include one or more multimodular enzymes. There is no article on fungal genomics that does not include an account of how many possible secondary metabolite clusters are present. A number of bioinformatic methods have been devised to detect secondary metabolite gene clusters [45]-[47]. Just one illustration of these possibilities is the identification of the gene cluster responsible for the synthesis of the first-line therapeutic agent pneumocandin in the genome of *Glarea lozoyensis* [48].

It is clear that the number of secondary metabolites that a fungus can potentially produce is much higher that those produced under laboratory conditions. Thus the availability of fungal genomes presents us with two challenges. Firstly, how do we activate the synthesis of a specific secondary biosynthetic pathway? Secondly, once we have produced a secondary metabolite, what is its biological activity? The activity we may detect in the lab is not necessarily the one which the fungus uses for its own unknown aims. Lovastatin is made by *Aspergillus terreus,* which I am sure does not care about the cholesterol levels of compulsive hamburger eaters, even if it may care about sterol synthesis of its ecological competitors. Suffice to say that in the Aspergilli, each of the early sequenced species carries in its genome 30–40 putative secondary metabolite biosynthetic gene clusters, and that there is not much overlap in the secondary predicted metabolomes in species of the same genus [49]. A similar situation is extant in the Fusaria and *Cochliobolus* species: among the former, *F. fujikuroi* could potentially synthesise secondary metabolites belonging to 45 different families. Of these 13–17 clusters involve polyketide synthases, but only three are common to all the *Fusaria* analysed [50],[51].

Activation of silent clusters could be obtained through overexpression of specific regulatory genes, which frequently can be identified because they are clustered with the biosynthetic genes. Deletion or mutation of broad domain regulators such as chromatin modifying proteins (erroneously called epigenetic methods) and/or modification of environmental conditions have also been used to de-repress the expression of secondary metabolite pathways. Among the latter, specific mention must made of co-cultivation methods, as pioneered by Brakhage and co-workers, in which a given fungus is co-cultured with other organisms with which it may interact in its (presumably) natural environment, that is to say obliging the fungus to care, to use the simile spelled above [52]. The reader is referred to the reviews of Brakhage and Schroeckh and Kim at al., [49],[53] for a detailed breakdown of these methods and for the identification of novel secondary metabolites in the Aspergilli.

While “omics” constitute a qualitative expansion in the possibilities of secondary metabolite identification, no similar revolution has occurred in methods to screen the biological action of an entirely novel natural product. While some screens such as antimicrobial activities, are straightforward, and can be trusted to robots, others are less so. Antitumor activity screening even if more laborious is possible using cell lines in culture. An entirely new metabolite may be an anti-depressant or a contraceptive, but we may never know, unless we use an adequate screen. It is interesting how a PubMed search yields more and more studies based on the use of plants and fungi in folk-medicine. In the absence of rational screen methods, this can be considered a reasonable, preliminary screen.

### Genome inspired therapies- pathogen genomics

Fungi are major plant pathogens. Some are strict specialists, such as *Ustilago maydis* (maize) or *Magnaporthae oryzæ* (rice), others such as *Botrytis cinerea* are much more eclectic in their choices. *Fusarium* species hosts range from cucumbers to humans. Not surprisingly, the importance of fungi as pathogens made them primary targets for whole genome sequencing. The life cycle and interaction with the host as some of these pathogens embody problems of basic biological importance and not surprisingly, those pathogens which were amenable to direct and/or reverse genetic techniques had already become models organism in their own right in the pre-genomic era. It is not possible to describe here how the availability of genomes and transcriptomes has changed the study of plant infections by fungi. As in other aspects of fungi biology, a shift from the specific to the global is taking place, in which it is possible to analyse the changes in gene activity of both the parasite and the host (Cairns et al. for a review dealing with both plant and human pathogen transcriptomes [54]). I will just pinpoint some somewhat arbitrarily selected examples of how the “omics” revolution is changing our way of studying fungal pathogens. The population structure of the pathogen is addressed by whole genome sequencing of different isolates, while RNAseq can be used to investigate the gene expression patterns of both pathogen and host, aiming to understand the mechanism of pathogenesis and the immune response of the host.

While a number of fungi are specific animal and/or human pathogens, the main public health concern has been the rise in opportunist pathogen infection in immunodepressed patients, the main culprits being *Candida* (mainly *C. albicans* and *C. glabrata*) and *Aspergillus* species (mainly *A. fumigatus*), but new species within and outside these genus appearing with increasing frequency. A recent review quotes a total of >2,000,000 life threatening fungal infections/year, with *Aspergillus*, *Candida*, *Cryptococcus* and *Pneumocystis* as the major worldwide opportunistic pathogens and with mortalities reaching varying between 20 and 90% [55].

In the nineteen century, infection by *A. fumigatus* was an exotic occupational disease, while it could be argued that at present the fungal opportunistic infections (with the notable exception of AIDS-related infections), including invasive Aspergillosis are mainly iatrogenic diseases, brought about by the use of immunosuppression in transplant patients. These high mortality figures depend both on diagnostic problems and on the inefficacy of antifungals, including the appearance of resistant strains. Of all the first-line antimycotic drugs, only the echinocandins target a specific fungal metabolic step, 1,3 β-glucan synthase. The hope is, that by identifying essential specific fungal genes, not present in the host, we should be able to design specific inhibitors. As a first step, one could try to establish a repertoire, within specific fungal genes, of those that are essential. Two different studies address this problem for *A. fumigatus*, one by heterologous transposition [56], the other by creating conditional lethals with the use of a regulatable promoter [57]. We are presented here with a similar problem to the one discussed above in relation with secondary metabolite synthesis. Finding essential genes is not difficult; the strategies used are an expansion at the genomic level, with the increased sophistication brought about by new technologies, of the searches for recessive lethal mutations carried on since the 1940s in *Drosophila melanogaster*. Another question is, once a fungal specific essential gene has been identified, to design an efficient inhibitor for the cognate protein product. This could be searched blindly using high-throughput techniques and/or rationally if we know something about the biochemistry and physiology of the protein we are trying to inhibit. As far as I know, no new potential antimycotic agent has yet been discovered through this strategy. A recent article addresses the possible differences detected in the relevant genomes between the cation channels of pathogenic fungi, and those of their hosts [58], but there is a long way from uncovering a primary sequence difference to designing of a specific channel inhibitor.

*Candida albicans*, the most frequent fungal pathogen, is a diploid. A haplo-insufficiency test has been devised, in which one allele is inactivated and heterozygous deletion mutants are screened for increased sensitivity to batteries of compounds. In principle all genes in the genome can be thus tested against any number of compounds [59]. Using a variation of this test, high throughput method and molecular modelling, a novel family of non-azole inhibitors of ergosterol synthesis was identified [60]. I have seen no publications following up these findings in experimental animal models.

Mucormycoses are relatively rare, but on the increase among immunodepressed patients, as secondary infections of severe wounds and also in patients treated for iron toxicity resulting from renal failure [61]. The genome of *Rhizopus delemar* shows an ancient whole genome duplication, followed by expansion of specific genes and the presence of four genes encoding spore coat homologue proteins (CotH), specific of the Mucorales. CotH proteins are ligands of GRP78, a chaperone which also can be localised at the surface of endothelial cells, also explaining the cell specificity of infection by members of this order [62]. While the association of CotH proteins with GRP78 was discovered by conventional co-precipitation methods, the knowledge that this association is limited to Mucorales, results directly from the availability of numerous genomes of that order. A recent review speculates as to whether the GRP78/CotH interaction could be a therapeutic target specific for Mucormycoses, a promising post-genomic possibility [61].

Emergent fungal diseases not only concern immunosuppressed humans. In recent years, widespread epizoonoses affecting wildlife have become pervasive. The causes of emergent zoonoses are not restricted to fungal pathogens, and whichever their immediate infectious cause, a crucial problem is to understand how the recent emergencies are connected with human activities leading to changes in ecosystems. If the fungal human opportunistic diseases are iatrogenic, the emerging fungal zoonoses are more generally anthropogenic, as environmental and climatic changes have been blamed for their recent appearance. Among the fungal agents, *Batrachochytrium dendrobatidis* (Chytridiomycota) is decimating frogs and toads while *Pseudogymnoascus* (*Geomyces*) *destructans* (Myxotrichaceæ) affect bats (see Eskew and Todd for a parallel of these emergent diseases [63]) and *Nosema* species (Microsporidia, see below) kills bees and has been blamed as a cause of colony collapse disorder (CCD), where worker bees suddenly disappear from a beehive [64]. Whole genomes are available for these pathogens and for the Chytridiomycota and microsporidia also for several other species of the cognate phyla.

Out of ~ 6000 extant species of amphibians 35% are menaced, while about 159 may already be extinct (http://www.iucnredlist.org/initiatives/amphibians/analysis). While the causes are surely complex, Chytridiomycosis is a major contributing factor. *B. dendrobatidis* was identified as a lethal frog pathogen as recently as 1998. Since then it has been reported world wide, affecting a wide variety of amphibian hosts. A sudden emergence of a new disease, affecting a wide variety of species implies either a sudden change in pathogen virulence (such as the acquisition of new genes by horizontal transmission, see below) or environmental factors which upset a previous pathogen/host equilibrium [65]. As for other pathogen host/interactions “next generation” genomics and transcriptomics have been used to investigate both the nature of the pathogen and the response of the host. A phylogeny, based on whole genome sequencing of 49 different samples of *B. dendrobatidis* shows that different lineages of the fungus long predated the emergence of the panzootic upbrake. One clade, the Global Panzootic Lineage, was seen as quite heterogeneous and emerging as long between 10,000 to 40,000 years ago [66]. The data are consistent with a scenario in which there has been no drastic change in the pathogen; but wide geographical distribution after (or coincident with) the onset of the panzootic outbreak.

### Fungal phylogeny and taxonomy

The idea that protein sequences could form the backbone of a new molecular phylogeny, is co-æval with the closing of what I have called the first revolution in molecular biology. In 1965, long before DNA sequencing became a reality, Zuckerkandl and Pauling proposed the concept of a molecular phylogeny based on protein sequences [67]. Molecular phylogeny was actually started even before the onset of DNA sequencing, by 5S and later 16S RNA fingerprinting, leading in 1976 to the three kingdoms of life proposal of Fox and Woese [68]. It took a few years before the sequencing of bacterial 16 S rRNA (and eventually eukaryotic 18 S) was established as a method of choice for molecular phylogeny. The transition from the one gene method to the present emphasis on whole genome-based phylogeny mirrors the transition from the second molecular biology revolution to the present genomic revolution.

**Figure 2:**
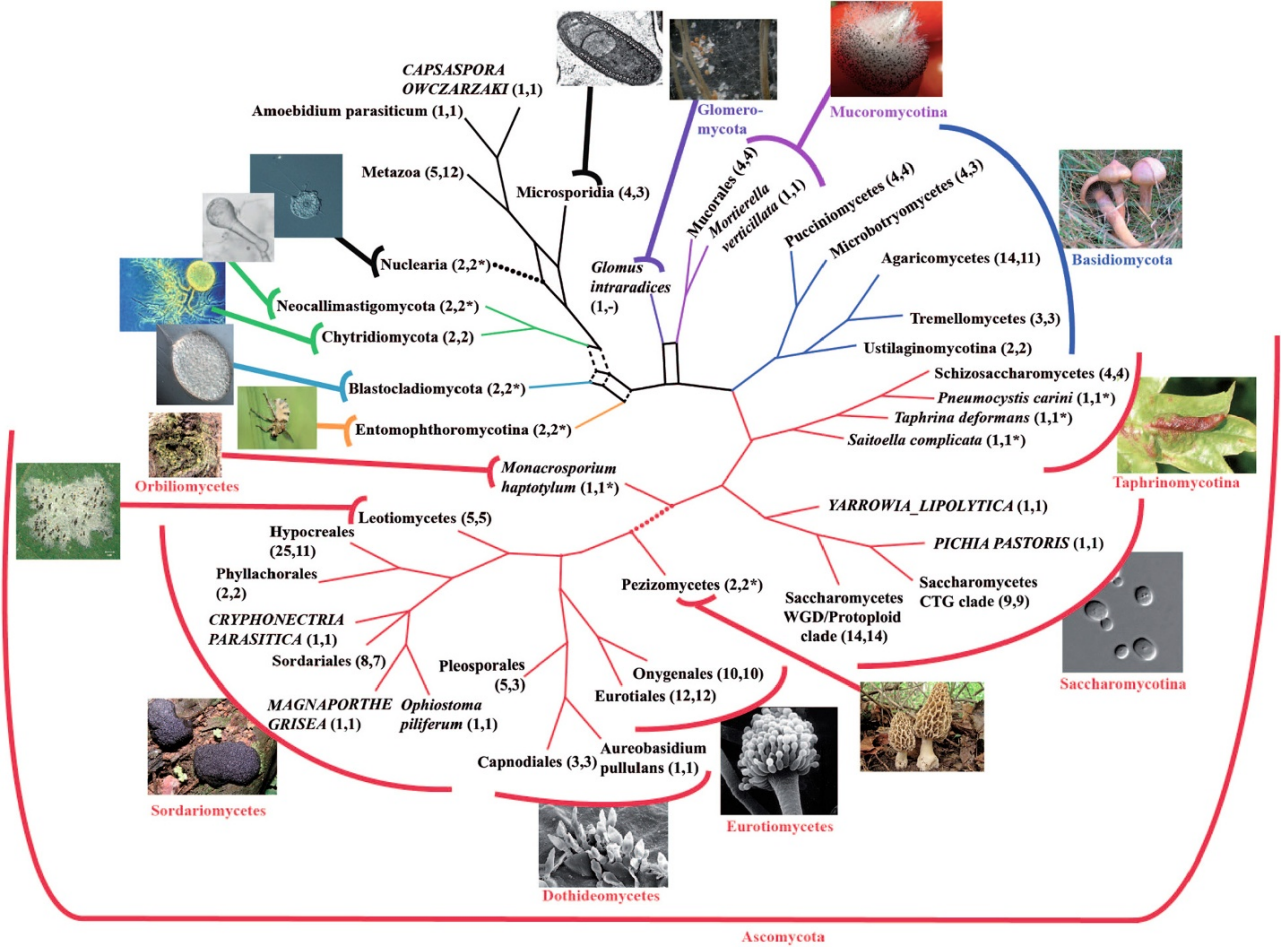
**A view of fungal phylogeny.** This figures is Sup Figure nine of Ref [72], obtained by whole genomic/ ESTs comparison (see text). For definition of the different data sets, see original article. The original legend is reproduced below. The phylogenetic backbone of the fungi based on 15 datasets. The numbers of species represented by each leaf are given in parenthesis for the data sets fungi_1 and fungi fungi_2, respectively. A *denotes those instances where either one or both species are absent from data set fungi_2 and are represented only in the supertree based on fungi_2A. A ‘-‘ indicates that a taxon is entirely missing in a data set. Colors highlight major systematic groups of the fungi (Ascomycota: red; Basidiomycota: blue; Mucoromycotina: magenta; Glomeromycota: purple; Entomophthoromycotina: yellow; Blastocladiomycota: marine; Chytriodiomycota/Neocallimastigomycota: green). Given the tentativeness in our reconstruction of the basal fungal relationships we keep the network structure for this part of the fungal backbone tree. Contractions of the dashed branches result in the topology that is suggested by our refined analysis of the early branching fungi with data set fungi_3.

Data derived from whole genome sequencing is now been almost routinely used to address phylogenetic problems from the kingdom to the species level, such as addressing the unresolved problems of the position of the Glomeromycotina and whether the “zygomycetes” are indeed monophyletic or a polyphyletic group (see for example Liu et al., 2009 [69]). I am aware of three studies that have addressed the phylogeny of the fungal kingdom through whole genomic data. The older study comprised 42 genomes and addressed mainly relationships among the ascomycetes, reflecting the availability of whole genomes at the time [70]. Later, an un-conventional methodology the “compositional vector method” was used to establish a phylogeny comprising 82 different completed genomes [71]. In the more recent study, 99 complete genomes and ESTs from 109 species were used to construct phylogenies [72]. Figure 2, taken from the above reference summarises the phylogenetic relationships within the fungi and of the fungi with their nearest sister phyla. Note the ambiguous placement of the microsporidia (which are fungi, see below), in relation to the nuclearia (which are surely not, but were not represented by whole genomes) is not resolved in this comprehensive study. Since the seminal work of Ebersberger et al. [72], a number of new genomes such as that of *Rozella allomycis* (which allowed placing the microsporidia, see below) have become available and more will in the near future. While this may not make much of a change in clades that are well represented, it may influence or resolve the positioning of others where only one or two species were extant at the time of the analysis of Ebersberger et al. [72].

*Encephalitozoon cuniculi,* a human pathogen, is a member of the microsporidia, which made it into the historical record as the second fungal species to be sequenced [73]. The taxonomical relationships of the microsporidia had been controversial for quite some time. Members of this phylum are obligate intracellular parasites of all metazoan phyla and even of some protists. In common with other widely different organisms (*Giardia lamblia, Trichomonas vaginalis, Entamœba histolytica*) microsporidia lack mitochondria. While it was speculated that these very diverse organisms represented a basal, “premitochondrial” kingdom of eukaryotes, called Archeozoa, it is now clear that in every single case recorded, the loss of mitochondria is secondary, and that structures akin to mitochondria are present (hydrogenosomes, mitosomes) and some typically mitochondrial genes reside in the nuclear genomes [74]. However, phylogenies based on single genes (such as those encoding HSP70 and tubulins) casted doubt on this placement, suggesting a relation to fungi, which was finally supported by the whole genome sequencing of *E. cuniculi* [73]. The placing of microsporidia as a basal group of the fungi involved two independent studies in which sequences derived in one study from 6 and in another case from 9 genomes, were compared by a variety of global methods with multiple genomes of fungi from other phyla [75],[76]. In the light of these studies whether we place microsporidia within fungi or outside fungi as a sister group may be a matter of taste, where we define somewhat arbitrarily what makes a fungus a fungus. However these quite robust phylogenies lead to another problem: if the loss of mitochondria (and other typical eukaryotic landmarks such a the Golgi, peroxisomes, 70S rather than 80S ribosomes, 9 + 2 microtube structures) is secondary, there must necessarily be organisms that are basal to both microsporidia and (other) fungi, unless by some unlucky event, these basal organisms are all extinct. A possible hint at the origin of microsporidia is its phylogentic clustering at the base of the fungal tree of life, with the only sequenced species of the Cryptomycota, *Rozella allomycis,* an obligate parasite of the water-mold *Allomyces* (Blastocladiomycota). This organism is the only member of a clade known through rRNA environmental sampling which can be grown in culture [77]. *R. allomycis* has mitochondria with a trimmed 12 kb mitochondrial genome. The published phylogeny is based on 200 genes, but it may be worth repeating this phylogeny using as an out-group a member of the nuclearia (supposed to be the sister group of fungi [69]) when a genome becomes available, and/or the already available *Fonticula alba*, a slime mould phylogenetically related to the nuclearia [78].

### The origin of specific fungal genes

A gene is “fungal specific” until new genomes reveal it in a non-fungal organism. In a preceding section, I have referred to XanA, the xanthine α-ketoglutarate dependent dioxygenase as a fungal novelty. In our original article we limited our searches to the then available ascomycetes and basidiomycetes [36]. The availability of genomes throughout the fungal tree of life could allow us to pin-point the node where the postulated gene duplication, which originated a *xanA*-like gene, occurred. A search on the species represented at the JGI revealed orthologous proteins, besides in the ascomycetes and basidiomycetes, in all the available genomes of the Mucoromycotina, but in no other fungal taxon. Two possibilities can account for this result, one is an ancient duplication of another α-ketoglutarate dependent dioxygenase encoding gene, the other being a horizontal transfer event from a non-fungal organism, both scenarios affecting a common ancestor of the Dikarya and the Mucoromycotina. A search in the NCBI database does not reveal any bacterial possible homologue, which could be at the origin of a XanA-like enzyme. This contrasts with another purine degradation enzyme, an alternative to the classical allantoicase, where horizontal transmission from bacteria to some fungi is almost certain [5]. However, strict orthologues of XanA, showing as much as 70% identity and with all crucial residues conserved are present in all sequenced strains of *Phytophthora parasitica*, in *P. infestans* and *P. sojæ* (see Figure 1, left panel) A maximum likelihood tree places the sequences of *Phyothphtora sp.* (only one included, Figure 1, right panel) *within* the Pezizomycotina rather that as an out-group, which strongly suggest an horizontal transmission from fungi to oomycetes rather than the other way around (see following section).

**Figure 3:**
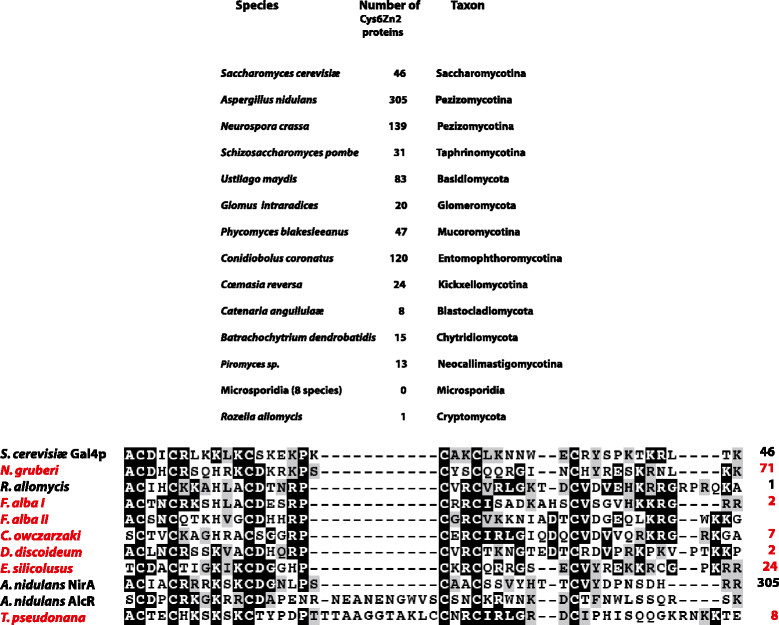
**Comparison of Cys6Zn2 in different organisms.** Top panel: Number of Cys6Zn2 transcription factors in representative species of different fungal taxa, or in same cases in the only available species of the taxon. Search carried out in the JGI fungal database (http://genome.jgi-psf.org/programs/fungi/index.jsf) with PFAM motif PF00172. Bottom panel: Alignment of a number of Cys6Zn2 motifs. Motifs corresponding to three well studied proteins (GAL4, NirA, AlcR) which bind to different DNA sequences are included. In red representatives of non-fungal Zn cluster proteins. For *F. alba*, the nearest sister species to the fungi available, the Zn clusters of both extant proteins are included. To the right of the sequence the total number of proteins of the species comprising canonical Cys6Zn2 clusters are recorded. Proteins which do not comprise all the conserved cysteines are not included in this count. Note that the homologue of *T. pseudonana* included has an extension in the third loop of similar nature to that of AlcR of *A. nidulans*. See text for complete names of non-fungal species. Searches carried out in JGI, NCBI, http://pfam.xfam.org/, alignment carried out with MAFT (G-INS-i) visualisation with Box-shade.

Zn-DNA binding motifs belong to several different classes. One, the nuclear receptor class, is unique to metazoans, and it has not been found even in the closest sister groups. Analogously the Cys6Zn2 (Zn binuclear clusters) are often quoted as exclusively fungal DNA binding proteins [79]. I investigated, using accessible databases, whether the Cys6Zn2 motif is present in all fungi. The results are shown in Figure 3 top panel, where the number of Cys6Zn2 containing proteins is shown for representatives of different fungal taxa. While no homologue is present in any of the microsporidia, just one is present in *Rozella allomycis*, the only sequenced representative of the Cryptomycota. I searched then the genome of *Fonticula alba*, the nearest relative of the nuclearia, the sister group to fungi, where I found two large proteins containing typical Zn binuclear cluster motifs (Figure 3 bottom panel). A search in http://pfam.xfam.org/ for motif *Zn_clus* (PF00172) lead to some surprises (some of these were reported previously [80]). Cys6Zn2 clusters are present in number of non-fungal organisms, some may make some sense while others do not. In *Capsaspora owczarzaki*, (Filasteria) an opisthokont, which belongs to a sister group of both fungi and metazoa [81], there are 7 bona fide Zn-cluster proteins. They are present in the Dictyosteliidea, with one or two Cys6Zn2 representatives/species. These findings are consistent with a scenario in which proteins carrying this motif were present in the base of the Unikonts (comprising opisthokonts, and amœbozoa, including Dictyosteliidea) and were lost in some taxa and expanded in others. The expansion of this protein family in fungi seemed to have happened at the base of the Dikarya. However other occurrences are more difficult to account for, such as the presence of one protein in two diatoms (*Thalassiosira pseudonana* and *Phaeodactylum tricornutum*, however the sequence is non-canonical in the latter species) and in a brown alga (*Ectocarpus siliculosus*). A number of proteins detected in *Hordeum vulgare* var*. vulgare,* and in no other plant should perhaps not worry us, they are typical fungal proteins, some showing as much as 89% identity with a protein of *Exophiala aquamarina* (Pezizomycotina) and are most likely due to fungal DNA contamination. But such trivial explanation cannot account for the 24 canonical Cys6Zn2 proteins present in *Ectocarpus siliculosus* or the 71 proteins carrying a Zn binuclear cluster recorded for *Nægleria gruberi* (Percolozoa, Heterolobosea), a fascinating organism which alternates between a flagellate and amoebic form (http://genome.jgipsf.org/Naegr1/download/Naegr_differentiation.mov) and which is phylogenetically as far from fungi as any other eukaryote could be. The *Nægleria* Cys6Zn2 proteins look quite different from their fungal counterparts, and present a variety of architectures. It looks like the organism took up the Cys6Zn2 finger motif and used it for its own ends. We would dearly like to know what these ends are. The expansion of Cys6Zn2 proteins in Dikarya is most likely due to their recruitment to regulate a diversified primary and secondary metabolism, including the ability to utilise the most disparate substrates as sole nitrogen and/or carbon sources. *Nægleria gruberi*, on the other hand is a predator who phagocytes bacteria, a style of life very different from that of saprophytic fungi.

### New insights into fungal evolution: horizontal transmission

The appearance of a typical fungal protein in diatoms and its expansion in *Nægleria gruberi* could be accounted for if Cys6Zn2 proteins existed at the root of eukaryotes, while suffering episodes of loss and expansion. An alternative scenario is that at one stage horizontal gene transmission has occurred between a fungus or a fungal ancestor and more remote organisms such as diatoms and a *Nægleria* ancestor. There are a number of such possible scenarios; the most parsimonious one would be the appearance of Cys6Zn2 proteins in an ur-unikont, followed by episodes of loss (including in the ancestor of metazoans) and expansion, with horizontal transfer to (some) diatoms and *Nægleria* species. Occam’s razor is not always an appropriate tool, thus any other scenario shuffling vertical and horizontal descent events can be envisaged.

It is arguable that the most fascinating concept arising from the availability of multiple genomes is the awareness of horizontal transmission between phylogenetically distant organisms as a motor of evolution. A new paradigm is being born, one in which organisms are not only related by descent, but also by common ecologies. Perhaps we should be looking at the Cys6Zn2 proteins of aquatic fungi, to find the nearest progenitors of the diatoms and *Nægleria* proteins.

In a simplified Darwinian view of the world, in which evolution is variation followed by natural selection, horizontal transmission is a drastic mechanism of variation, in which a ready-made gene or group of genes is inserted into an alien genome, creating a whole new series of possible interactions at all levels, from the transcriptome to the metabolome to community ecological interactions. Such an occurrence may be at the root of the appearance of new pathogens [82].

In the prehistory of the concept of horizontal transmission, Peñalva and co-workers [83] (see Brakhage et al. [84] for a more recent account) proposed that ability to produce β-lactam antibiotics was transmitted twice independently from *Streptomyces* species to fungi. Since then, horizontal transmission of secondary metabolite genes has become a household concept in fungal biology (see below).

While some instances of transfer from bacteria to fungi were discovered incidentally, a whole genome approach revealed 713 transferred genes from bacteria to fungi, derived from a minimum of 235 individual events. These events affected mostly the Pezizomycotina, but all analysed groups were affected, including Saccharomycotina, Taphrinomycotina and Basidiomycota, and more basal groups such as Microsporidia, Chytridiomycota and Mucoromycotina. Particularly interesting is the fact that more than one transmission event affected similar genes or pathways, genes encoding an arsenate detoxification enzyme were independently transferred to Y*arrowia lipolytica* (Saccharomycotina) and to *Rhizopus oryzae* (Mucoromycotina), catalase and amino acid racemase genes were multiply transferred to different phylogenetically distant species [85].

Reports of intra-kingdom transfer of the biosynthetic clusters encoding secondary metabolism biosynthetic genes are not yet a flood but rather a steady flow. The synthesis of fumosisin apparently travelled from a member of the Sordariomycetes to *Aspergillus niger*, [86] it also jumped about among the *Fusaria* [87]. Intra-fungal travel of genes have been summarised by Richards [88],[89]. The latter review confirmed 323 bacterial transfers to fungi and 9 events of intra-kingdom transfer, including genes involved in both primary and secondary metabolism.

The biosynthesis, considered as restricted to some Aspergilli, of the related metabolites aflatoxin and sterigmatocystin is arguably the most thoroughly studied secondary metabolic process [90]. A gene cluster encodes all enzymes involved in the synthesis together with the specific transcription regulatory gene *aflR* (∼54 kb, 23 genes in *A. nidulans* which produces sterigmatocystin, ∼67 kb, 26 genes in *Aspergillus flavus* which produces aflatoxin). A whole genome survey, involving 94 species has revealed the presence of an orthologous cluster in *Podospora anserina*, but not in other sequenced Sordariomycetes The *A. nidulans* and *P. anserina* clusters show quite striking intra-cluster synteny [91].

Dothistromin, another secondary metabolite chemically related to aflatoxin and sterigmatocystin, shares with the latter a common intermediate. Dothistromin is produced by the pine pathogen *Dothistroma septosporum* and a few other Dothideomycetes. Results of Bradshaw et al. [92], together with those of Slot and Rokas 2011 suggest a story of horizontal transfer of the whole cluster from an ancestor of *A. nidulans* to *P. anserina* and independently to an ancestral member of the Dothideomycetes followed by episodes of cluster fragmentation with recruitment of additional biosynthetic genes. Sterigmatocystin production has been detected in other widely diverse ascomycetes [93], thus other episodes of horizontal transmission of the cluster may have occurred. However, there is another possible extreme scenario involving only vertical transmission from an ascomycete ancestor followed by multiple loss and fragmentation. A number of arguments make this scenario highly improbable for the *A. nidulans*/*P. anserina* orthologous cluster [91].

Evolutionary stories as just stories (some would say “just so” stories), and a number of combinations of horizontal and vertical transfer episodes are possible, which may be become more or less improbable when a comprehensive phylogeny of many diverse aflatoxin/sterigmatocystin/dothistromin biosynthesis occurrences is undertaken.

There is no *a priori* reason why horizontal transfer should be limited to bacterial-fungal and fungal-fungal transfer. While the numbers of plant genomes available increase, so do the opportunities to investigate plant-fungal gene transfers. This is a matter of some interest, given the large number of fungal plant pathogens and symbionts. An automated comparison of 6 plant, species and 46 fungal species resulted in only 9 strong candidates for gene transfer between plants and fungi of which 5 were from fungi to plants and 4 from plants to fungi. Two of the fungal genes transferred to plants possibly originated from prokaryotes. This study was necessarily limited by the genomes available at the time. In particular, only three genomes outside the Dikarya were available. Only one ectomycorrhizal fungus (*Laccaria bicolor*) could be included. The genome of *Glomus intraradices*, an arbucular mychorrrizal fungus, was not yet available. Within these necessary limits the authors conclude that plant/fungal transfer events are rare and ancient [94]. One can look forward to a newer survey in which a number of basal fungal species now becoming available is included.

Ernest Rutherford is famously supposed to have said, “All science is either physics or stamp collecting”. Molecular biology can be construed to represent a transition from stamp collecting to physics. Genome gazing, however sophisticated the bioinformatic methods we may use, suspiciously resembles a reversion to stamp collecting. We have collected a number of instances of intra and inter-kingdom horizontal gene transfer events. Perhaps we could say, that there is nothing wrong with stamp collecting in biology and that all science starts with or goes through stamp-collecting phases.

While we can speculate on the evolutionary importance of horizontal gene transfer, we are in the dark regarding the mechanism of such transfer in eukaryotes, more strikingly so in organisms such as fungi which acquire nutrients through extracellular rather than intracellular digestion. How does intact DNA get in? How and how often does it escape nucleases? How does it go through the nuclear membrane? Evoking transposons only displaces the problem, as to how a transposon carrying a given gene travels from one organism to the other. Phylogenomics tells us that horizontal transfer even if not rampant is much more than a naturalist curiosity. It opens a whole new field of enquiry pertaining to the mechanism(s) of inter-organismal DNA mobilisation.

### New insights into fungal biology: gene clustering

I have worked for a good part of my scientific career on two primary metabolism gene clusters of *A. nidulans*, the nitrate assimilation gene cluster [2],[95],[96] and the proline assimilation gene cluster [97],[98], while initiating the work on the *alc* gene cluster, then continued by Betty Felenbok and co-workers [99]. A situation diametrically opposed to clustering is found for the purine utilisation pathway of *A. nidulans* where none of the 17 genes encoding enzymes or transporters of this pathway is clustered with any other [100]. I have always wondered why the nitrate assimilation genes are clustered in *A. nidulans* and dispersed in *N. crassa* and the proline assimilation genes are completely clustered in *A. nidulans* and dispersed in *S. cerevisiæ* and why we see within the same organism clustering in some catabolic pathways and not in others. Our ability to interrogate a large number of genomes may be giving some insights into these old questions.

In the previous section I have mentioned that secondary metabolites genes are usually clustered. An attractive idea is that these clustered genes share a common chromatin organisation [101]. Chromatin proteins and chromatin modifying proteins have an important role in secondary metabolism gene expression [90],[101]-[104], however evidence for a specific chromatin (or heterochromatin) specific structure of secondary metabolism clusters is wanting. Many secondary metabolism gene-clusters are located in sub-telomeric positions [105], but we really do not know whether they are subject to sub-telomeric heterochromatic silencing of the type described for *D. melanogaster* or *S. pombe* [106]. The attractive simple model of facultative heterochromatisation of secondary metabolism gene clusters during vegetative growth, for which I am partly responsible, may well be an oversimplification.

It has been proposed that clustering of secondary metabolite genes is a result not of selective pressure arising from the necessity of co-regulation, but rather that the whole cluster behaves like a selfish DNA segment that persist through horizontal transmission [107], even if we have no hint why some DNA segments may be more prone to horizontal transmission than others. Necessarily, once a cluster is transferred, another level of selection acting on the phenotype of the whole organism will be operating. But this second level of selection only cares about the selective value of the metabolites resulting from the pathway and eventually about their toxicity (see below).

To borrow a terminology from linguistics, when discussing gene clustering two types of explanations are possible: diachronic (historical) explanations, concerned with the origin of the cluster and synchronic (functional) explanations, concerned with its expression and regulation here and now. A paradigmatic example of a diachronic explanation is that of Wong and Wolfe in “Birth of gene cluster by adaptive gene relocation” [108]. Clustering of six genes involved in allantoin utilisation is demonstrated to be a relatively recent novelty appearing at one specific stage of the evolution of the genus *Saccharomyces.* This novelty coincides with the ability to grow in anaerobic conditions and with the inability to utilise urate as a nitrogen source (a process that generates reactive oxygen species), due to the concomitant loss of the genes encoding urate oxidase and the urate/xanthine transporter [108],[109]. Not surprisingly three other genes (orthologues of *xanA*, *uaX* and *uaW* of *A. nidulans*, [100]), also necessary for xanthine and urate utilisation, are lost together with the appearance of the allantoin utilisation cluster (my own unpublished observations). An allantoin specific transporter gene, *DAL4* , integrated in the cluster, originated from a duplication of the uracil transporter gene *FUR4*, concomitantly with the birth of the cluster [108].

The clustering of three genes involved in nitrate assimilation (encoding the transporter, nitrate reductase, and nitrite reductase) in a number of fungi (including Eurotiales among the Ascomycetes and at least some Basidiomycetes) but not in others, has been interpreted as a result on horizontal transmission of the whole cluster from an Oomycete to the ancestor of Dikarya or perhaps even earlier, as the genes (but not as a cluster) are present in Mucoromycotina [110]. Episodes of de-clustering would have repeatedly occurred among the Dikarya.

**Figure 4:**
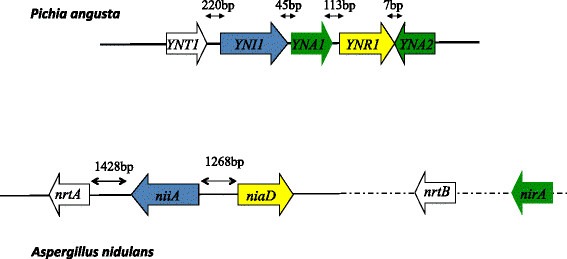
**Comparison of the nitrate assimilation gene cluster in** ***A. nidulans*** **and** ***P. angusta*** **.** White, nitrate transporter, yellow nitrate reductase, blue nitrite reductase, green transcription factors. In *A. nidulans* a second transporter gene (*ntrB*) and the *nirA* transcription factor gene are in the same chromosome (VIII) as the gene cluster but not genetically linked to it or to each other.

The assimilation of nitrate has been studied in detail in three ascomycetes, *A. nidulans* [2],[95],[96] *, N. crassa* [111] and a member of the Saccharomycotina, *Pichia angusta* (*Hansenula polymorpha*). In the latter organism the genes of the nitrate utilisation pathway are completely clustered. This cluster comprises not only the three genes mentioned above, but also two Cys6Zn2 transcription factors (Yna1 and Yna2 [112]), which are different from the orthologous pathway-specific *A. nidulans* NirA and *N. crassa* NIT4 transcription factors. The regulatory patterns of *A. nidulans* and *N. crassa* are very similar, not withstanding the fact that no clustering is extant in *N. crassa*. Figure 4 compares the clusters of *A. nidulans* and *P. angusta*. I can see no obvious explanation for the assimilation of two novel transcription factor genes into the cluster of *Pichia angusta*. While horizontal transmission is a suitable explanation for the ancestral presence of the cluster, there is no clear rationale either for de-clustering or for assimilation of new genes into the cluster. The cluster has been functionally characterised in another member of the Saccharomycotina, *Arxula* (*Blastobotrys*) *adeninivorans*, where it includes two transporter genes but not the transcription factor genes [113]. *Arxula* is a basal clade of the Saccharomycotina [114], which supports a secondary clustering of the regulatory genes occurring after the divergence of *Arxula* and *Pichia*. Complete clustering of the nitrate assimilation pathway is found in yet another nitrate utilising member of the Saccharomycotina, *Kuraishia capsulata* [115]. The cluster includes two regulatory genes, which are however surprisingly different from Yna1 and Yan2, and this in spite of the phylogenetic proximity of the two species. A search in the genome of *A. adeninivorans* failed to find any possible orthologues of Yna1, Yna2, the two regulatory genes from *K. capsulata* or NirA/NIT4. It will be interesting to know which transcription factor(s) has been recruited to regulate this pathway in *A. adeninivorans*.

Rokas and co-workers suggested a different evolutionary rationale for clustering [116]-[118]. They propose that clustering of the primary metabolism genes of fungi occurs when one of the products of the metabolism is toxic. This is the case for galactose utilisation, where Galactose-1-phsophate is toxic, and clustering of three enzymes of galactose metabolism has occurred independently twice within the Saccharomycotina, and once in the basidiomycetes, with a probable horizontal transfer from a *Candida* species to *Schizosaccharomyces* [116]. Tyrosine utilisation as nitrogen source involves the very toxic intermediate fumarylacetoacetate, The production of betaine from choline also involves a toxic intermediate (betaine aldehyde). Clustering is seen in all these pathways. I could add that ∆^1^ pyrroline-5-carboxylate, the product of proline oxidation, is converted non-enzymatically to glutamic semialdehyde which is highly toxic [119], and nitrite, the product of nitrate reduction in the nitrate assimilation pathway is also toxic [120] (see above, the cognate genes in these pathways are clustered in some organisms but not in others).

The selective advantage of clustering is embodied by the fact that if the genes are clustered, the probability of loosing just the one gene encoding the enzyme responsible for the detoxification of a toxic intermediate becomes automatically lower. If the cluster is lost as a whole, a catabolic pathway is lost but no toxic intermediate is accumulated. The selective advantage resides in this “all or none” situation. However there are counter examples: uric acid, allantoin and allantoic acid are highly toxic in *A. nidulans* [121] and as mentioned above, no clustering is seen for the genes of the purine degradation pathway in this organism [100]. It can be argued that selective pressures differ from one organism to another, from one pathway to the other. If we want to avoid a circular argument, we need independent, ideally experimental evidence, pertaining to these proposed different selective pressures.

I think this discussion illustrates both the virtues and limitation of the genome gazing approach: it can surely suggest correlations, which may be supported or disproved by additional examples. It should lead to evolutionary experiments, to check if under challenging conditions “birth of a cluster” or “de-clustering” occurs. Work based on genomics seems to be excellent to generate diachronic hypotheses; we also need experiments to access the synchronic level of explanation.

### Whither the model organism?

*Definition of model organism: The organism on which I work, with the exclusion of other organisms, principally those used by others to address the same biological problem as I do.*

The concept of the model organism implies a societal positive feedback process, by which an organism is chosen to investigate a given problem because of specific characteristics or accessibility to experimental manipulations; this generates a research community, whose coherence and number strengthens the “model” status of the chosen organism. *Drosophila melanogaster* was chosen by Morgan as the organism to construct classical genetics, but both Beadle and Ephrussi abandoned it (for *Neurospora crassa* and *S. cerevisiæ* respectively) as unsatisfactory to identify the primary product of the gene. The work of Beadle, associated with Tatum, lead to the one gene-one enzyme concept [10] and collaterally established *N. crassa* as a fungal model organism.

The crowned king of fungal model organisms is with no question *S. cerevisiæ*. I have hinted in the first section of this article at some of the reasons for this status. It was the first eukaryote where reverse genetics was possible, which in turn resulted in a research community numerous enough to afford to carry out the first eukaryotic whole genome sequencing, followed by systematic gene inactivation and GFP (green fluorescent protein) tagging, not forgetting the creation of a species specific data base, thus further reinforcing the model organism status.

One of my favourite recent articles describes a self-sustaining, albeit unstable mechanism of gene silencing in *Mucor circinelloides* [122]. Whether *M. circinelloides* is a model organism or not is possibly a matter of taste. Perhaps now it is becoming one. Inspired genome gazing, with or even without the assistance of sophisticated bio-informatics can lead to new discoveries, which then have to be tested in the appropriate organisms. For example, comparing by hand, or better to say by eye, gene models of a biotin biosynthetic gene in different fungi my colleague Michel Flipphi realised that canonical splicing of some introns would lead to inactive, frame-shifted proteins. He then realised (again by eye) that there were inner introns interrupting the donor sequence of the main intron. The experimental test for introns within introns (stwintrons, splicesosomal twin introns, a new concept) was carried out necessarily not in our life-long model *A. nidulans,* but in *Fusarium verticilloides*, *Trichoderma reesei* and *Botrytis cinerea* [123].

It is clear that model organisms are here to stay. It would be superfluous to attempt to replicate in other Aspergilli the wonderful work of Peñalva and collaborators on the Golgi apparatus of *A. nidulans* [124]. Nevertheless caution is required when extrapolating findings of one organism to the other, even within the same genus as recent findings demonstrate [125]. We need to work with related organism, to understand morphological differences such as why the conidia of *A. nidulans* and *A. fumigatus* are uni-nucleate and those of *A. niger* multinucleate, why the conidiophores of *A. nidulans* are biseriate and those of *A. fumigatus* uniseriate. Not withstanding the advantages of *A. nidulans* as a model, if we want to understand why *Aspergillus versicolor* can grow in the Dead Sea and thrive at pH 9.0 we have no alternative but to work directly with *A. versicolor*.

The present post-genomic revolution is creating almost limitless opportunities to initiate new work in a large variety of organisms. Search of databases, driven by a knowledge of biological and biochemical processes leads almost fatally to organisms far beyond the restricted word of “models”. Indeed we have more genomes than people able to work with the cognate organisms.

I have mentioned in the first section of this review how the “second revolution” (gene cloning, reverse genetics, limited sequencing) transformed the practice of our science. Post-genomic biology will necessarily lead to sociological changes. On the one hand we start sharing with experimental physicists the experience of publishing articles with over one hundred authors. On the other hand, the existence of extensive public databases allows individual isolated scientists to ask specific questions, if they have a clear biological problem in mind. In the course of writing this review some new questions arose and all I had to do was to address the appropriate databases and online calculation facilities. We could say that the lonely scientist pottering away on his/her computer has a parasite/host relationship with publically funded databases. The NIH and the USA Department of Energy fund the databases I mostly use, thus I am being subsidised unwittingly by the North American taxpayer. While I may consider that this is a just return for the European and Latin American brain drain (these are the ones that concern me directly), it also means that political changes or budgetary considerations can jeopardise the existence of major scientific facilities. This is one problem we have to face and solve in this post-genomic era.

The post-genomic era could lead to scientific hyper-autism or to new convivial networks. I am lucky to have a number of former students, post-docs and new colleagues with whom to share and discuss my genomic gazing and I wish to thank all of them warmly for the fun we had in these few years since my official retirement.

### Concluding remarks

This already lengthy review is necessarily incomplete. There are a number of aspects of fungal biology, which surely can be or have already been illuminated by “omics” which I did not address. I try to indicate them below. In all fungal genomes, there are orphan genes. Those come in two flavours, those for which we have no inkling of their function, and those who belong to characterised gene families, but where we ignore the specific function. Of the 300 odd Cys6Zn2 proteins of *A. nidulans* we only know the function of a few. Alternative splicing is present in the fungi, and it has already been investigated genome and transcriptome-wise [126]-[128]. Metagenomics is usually a pursuit of bacteriologists, but it is starting to be extended to fungi [129]. Related to this question is whether the concept of the pangenome is relevant to fungi [130]. The availability of complete genomes has revealed mating type genes in many fungi supposedly asexual, leading in some specific cases to experimental verification of sexuality [131]-[133]. Transposons of different classes are present in all fungi but their distribution is patchy, and this patchiness in striking for helitrons ([134] and my unpublished observations). An investigation of their distribution may be quite relevant to the mechanism of horizontal transmission. Prions and a number of epigenetic phenomena, which are formally prion-like, have been investigated in *P. anserina* and *S. cerevisiæ* [122],[135],[136]. May genomes and transcriptomes help reveal the existence of new prions [137] or prion-like phenomena? Perhaps the theme I regret the most not having included is the use of “omics” to investigate community relations between organisms. These go from inter-organism signalling, even among organisms of different kingdoms [52], to the investigation of the dynamics of mycorrhizæ [138] and to the challenging symbiosis of fungi and algae on lichens [139]. The latter is now opened with the completion of a number of genomes, in one particular case of the genomes of both partners.

### Endnote

^a^Part of this effort was carried out in Hans Kuntzel’s laboratory in Gottingen. I take the opportunity to stress the contribution of Richard Waring and Terry Brown, and the forwardlooking leadership of R. Wayne Davies to this effort. Very few remember today that it was Wayne who first planned the complete sequencing of Chromosome III of *S. cerevisiæ*.

## Authors’ original submitted files for images

Below are the links to the authors’ original submitted files for images. Authors’ original file for figure 1
Authors’ original file for figure 2
Authors’ original file for figure 3
Authors’ original file for figure 4
