# Supplementary figures and images for: Fungal biology in the post-genomic era
Source: Fungal Biol Biotechnol. 2014 Oct 14;1:7. doi: 10.1186/s40694-014-0007-6 (PMC5611559; doi:10.1186/s40694-014-0007-6)

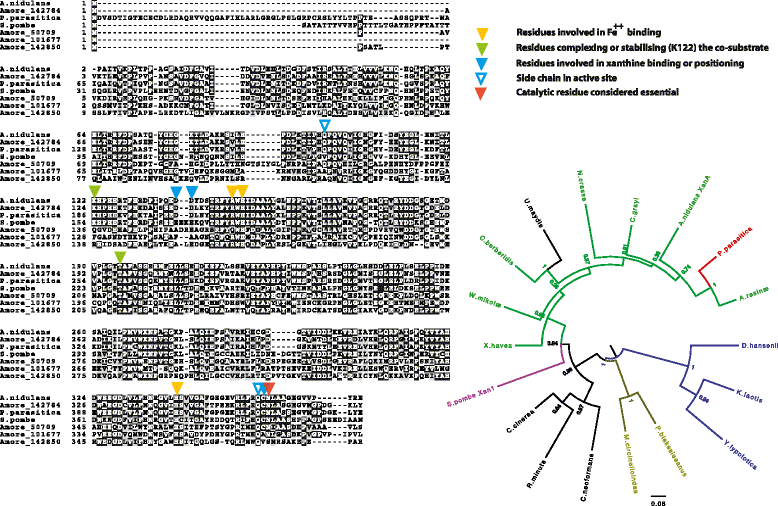

Supplement: Supplementary file 1 — Authors’ original file for figure 1 [file 40694_2014_7_MOESM1_ESM.gif]

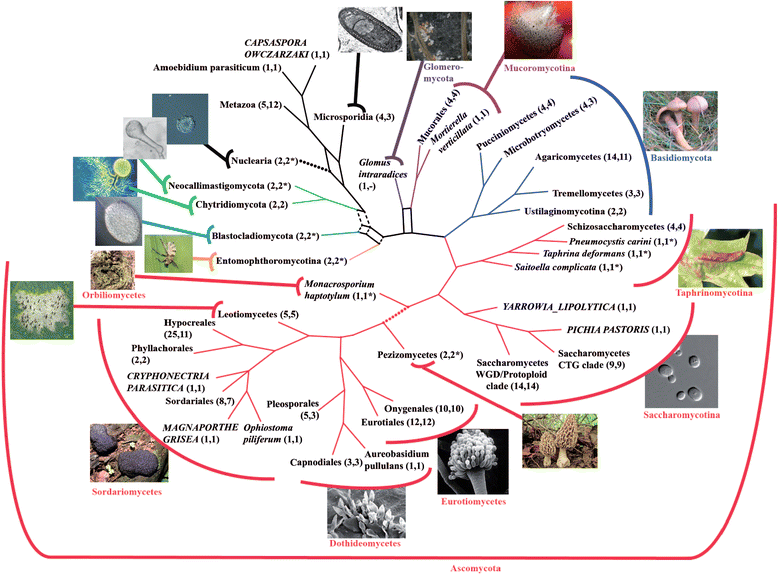

Supplement: Supplementary file 2 — Authors’ original file for figure 2 [file 40694_2014_7_MOESM2_ESM.gif]

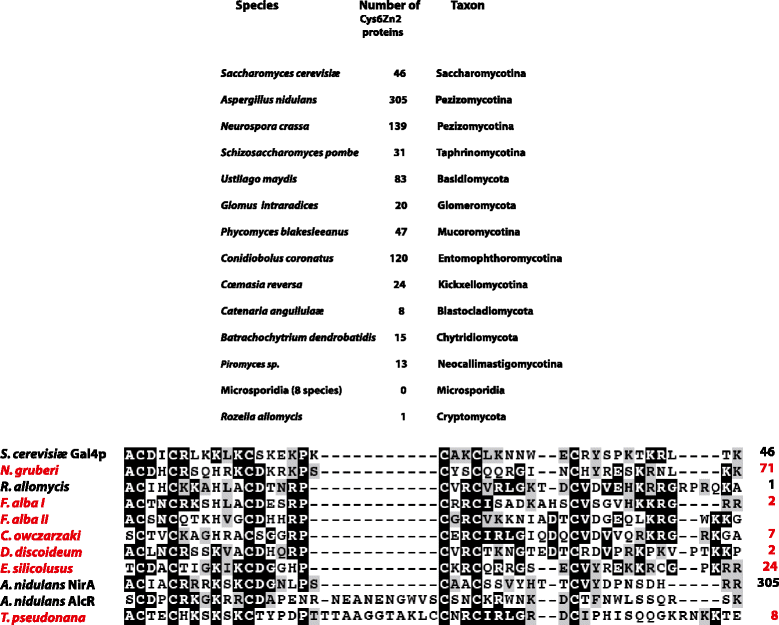

Supplement: Supplementary file 3 — Authors’ original file for figure 3 [file 40694_2014_7_MOESM3_ESM.gif]

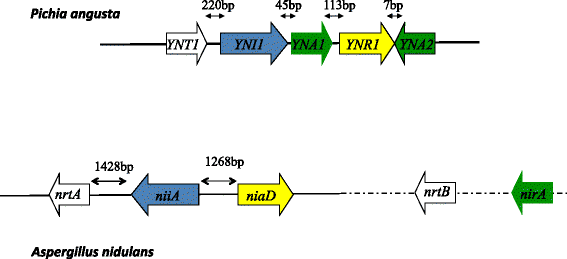

Supplement: Supplementary file 4 — Authors’ original file for figure 4 [file 40694_2014_7_MOESM4_ESM.gif]
